# Correspondence

**Published:** 1869-05

**Authors:** F. K. Bailey

**Affiliations:** Knoxville, Tennessee


### Correspondence.

KNOXVILLE, TENNESSEE, *April 21, 1869.*

PROFESSOR DAVIS:—The weather since last fall has been mild. Only during the month of December was it cold, as that term is used in this latitude. At one time the mercury went down to 5° above zero, and a little snow fell. At no time during the whole winter has the ground been covered with snow.

There was no sickness worth mentioning, till, in January, when measles began to prevail. In February, the disease became epidemic; and whole families were prostrated at one time. Most cases were of a simple type, during the month of February, but, in March, was observed, in many cases, a tendency to bronchial and pneumonic complications, with sore-throat and a continued type of fever. Intestinal worms, in a few cases, figured quite prominently, in connection with measles. I may say in this connection, that children are more affected by worms here than I had found to be true in Michigan or Illinois.

February 21st, I was called to see a boy of 14; light complexion and hair.

Besides finding the usual indications of measles, ascertained that for some months previous he would scream at night, and get up in a condition of somnambulism, at times uncontrolable. The tongue was covered towards the base with a thick, creamy coat, but red and pointed at the tip. Although the indications of measles were plain, still, I diagnosed worms to be an immediate cause of the noisy demonstrations during sleep. Prescribed hyd. cum. creta, gr. x.; pulv. Doveri, gr. xij.; santonin, gr. viij.; M. F. pulv. No. 4, sig., one every three hours, to be followed with castor oil and oil turpentine.

Calling next morning, found that a great number of worms (lumbricales) had been voided from the rectum, besides vomiting some, and even two or three had crawled from his mouth while asleep. To take pulv. Doveri, P. R. N.

23d.: *10 A.M.* Found eruption fully developed upon the face and neck, and the usual catarrhal symptoms. Ordered teaspoonful doses every four hours, of the following mixture:—

℞. Spts. Nit. Dulc, ------------------------------ ℥j.

Syr. Ipecac, } āā. ------------------------- ʒiij.

" Scilla, }

Tr. Sang. Canad. ------------------------ ʒij.

This boy continued to void worms for some days; and the wild demonstrations gradually ceased to occur. Convalescence seemed established, but, March 11th, he was attacked with pleuro-pneumonia upon the right side. Expectorants and blistering were resorted to, and in a week or more he was about again.

March 2d. Called to attend a boy of 13, laboring under the usual prodromic signs of measles. From some of the symptoms, suspected worms; and, accordingly, gave santonin, with hyd. cum. creta; with the effect to bring some eight or ten away. The eruption came out in due time; and he recovered without any complications.

One case occurred, superadded to common, continued fever. A little girl of 7, with light skin and flaxen hair, was taken, March 8th, with a convulsion. Was called immediately, and found that the fit had subsided; but reaction was coming up rapidly. The child had complained of slight indisposition for two or three days; tongue coated; skin dry; and urine high colored. The fever continued steadily from day to day, when, on Friday, the 12th, the eruption of measles came out finally. The usual catarrhal symptoms were present, but not severe. The fever progressed steadily through the month; the pulse running as high as 140 in a minute, with hot skin, scanty and very high colored urine; tympanitic abdomen about the 21st day, with profuse and very offensive stools. The tongue was very red and clean; appetite wanting; emaciation and extreme debility. The treatment was mainly expectant, the indications from day to day suggesting the remedies.

To-day, April 7th, she is improving finely.

April 14th. Was called to see a child of a year old, that had just recovered from a mild form of measles, laboring under capillary bronchitis. The secretion was so copious as to almost produce suffocation; the difficulty of expectoration being enhanced by the very ropy mucus. By the use of small doses of hyd. cum. creta and pulv. Doveri, alternated with syr. ipecac and tr. ginger, continued two days, the mucus became more liquid, the discharges from the bowels very copious, and all the suffocation symptoms relieved. The tendency to such relapses seems to be caused by an excess of fibrin in the blood, which is obviated at once by the use of mercury and other antiphlogistic remedies. The difference between this locality and that of the valley of the Mississippi is very noticeable, especially to one who had for many years observed diseases in no other region of country.

The air upon this elevated range is always pure, and the depressing effects of miasma wholly unknown. Convalescence from attacks of disease seems to be rapid, and health soon restored. Relapses are more uncommon, from the fact that the *vis medicatrix naturœ* has less to contend with.

I will conclude by giving the following description of cases coming under my observation:—

BELL'S PARALYSIS.

March 23d, 1869. A man called at my office laboring under facial paralysis. The left eye was wide open, and could not be closed. On winking the right eye, the left remained unmoved.

He could neither blow, whistle, nor spit. In the act of laughing, the left side of the face was not moved, giving quite a ludicrous appearance.

On opening the mouth, his features were awry. On examining the throat, the arch formed by the pillars of the fauces was larger on the affected side, the uvula inclining to the sound side. Articulation somewhat deficient. The eyeball perfect in its movements. Slight difficulty in mastication, with an inability to move the morsel from the left cheek. Slight deafness in the left ear.

The affection was first noticed after riding in a railroad car, with the left cheek exposed to a current of air from an open window. I have had no opportunity as yet of attempting a cure.

HERNIA OF UNUSUAL SIZE.

Charlotte Freeman, mulatto, *æt.*, about 35, lived with her master in S.W. Virginia till the fall of 1863, when she came to this place. Last summer she consulted me in reference to a "rupture." I found an immense left inguinal hernia, reducible, but not capable of being kept up, by reason of the size of the sac. Advised the use of a suspensory apparatus; and saw no more of her till a few days ago. I was called to prescribe for her child, and took occasion to obtain a history of the case.

At about the age of 20, she first noticed a tumor in the left groin, about as large as a hen's egg. Her master procured a truss, which enabled her to keep about without any trouble. When it was worn out, her master refused to get another; and soon it began to enlarge. When the war commenced, she says it was about as large as a "pint bowl."

During the war, a kick from a mule at one time, and from a cow, while milking, at another, made her much worse.

She says, the last mentioned accident brought it to the present size, some idea of which will be conveyed by the following measurements:—

Circumference at the largest point 20 inches. From the upper margin of the ring around the tumor, longitudinally, to the opposite side or edge of the ring, 24 inches. The most dependent point of the tumor is four inches above the patella; and the woman is fully the average in height. The tumor is egg-shaped, with the larger end downwards.

On lying down, the whole contents of the tumor will readily return to the abdominal cavity, except what appears to be a portion of omentum adherent to the sac, anteriorly. This portion causes an appearance of bulging, and it looks as though one or more of the internal coats of the sac had been ruptured. This was caused, she says, at the time of receiving the blow from the cow. When the intestinal contents of the sac are within the abdomen, the hypertrophied integuments and this supposed omental adhesion make a mass as large, nearly, as a cocoanut. This prevents a truss being worn; and there is no way for the poor woman to do when in the erect position but to let the whole hernial mass *swing*. She goes about and labors hard, with but little apparent inconvenience. Both rings seem merged into one large opening about two inches across.

She has had nine children, four of them since the tumor attained its present size.

F. K. BAILEY, M.D.

